# Strengthening digital health local capacity: driving forces and milestones in Ethiopia

**DOI:** 10.1093/oodh/oqaf024

**Published:** 2025-10-03

**Authors:** Amanuel Biru, Wubshet Denboba, Dawit Birhan, Abebaw Gebeyehu, Gemechis Melkamu, Tewodros Kersie, Oli Kaba

**Affiliations:** Digital Health, JSI Ethiopia Country Office Sunshine tower #4, Airport road, Addis Ababa, Ethiopia; HIS and Data Use, JSI Ethiopia Country Office Sunshine tower #4, Airport road, Addis Ababa, Ethiopia; Digital Health, JSI Ethiopia Country Office Sunshine tower #4, Airport road, Addis Ababa, Ethiopia; M&E and Research, JSI Ethiopia Country Office Sunshine tower #4, Airport road, Addis Ababa, Ethiopia; Digital Health LEO, Ministry of Health of Ethiopia Sudan St, P.O. Box 1234, Addis Ababa, Ethiopia; Digital Health LEO, Ministry of Health of Ethiopia Sudan St, P.O. Box 1234, Addis Ababa, Ethiopia; Digital Health LEO, Ministry of Health of Ethiopia Sudan St, P.O. Box 1234, Addis Ababa, Ethiopia

**Keywords:** Ethiopia, digital health, health information systems, governance, interoperability, workforce capacity, sustainability, LMICs

## Abstract

**Background:**

Strengthening local capacity is central to building sustainable digital health systems in low- and middle-income countries (LMICs). Ethiopia’s experience illustrates how nationally led strategies can drive digital transformation.

**Methods:**

This study uses a qualitative descriptive case study approach, drawing on government policies, program reports, peer-reviewed literature and the authors’ direct implementation experience. Data were reviewed and analyzed thematically to trace the driving forces, milestones, achievements and challenges of Ethiopia’s digital health journey between 2016 and 2025.

**Findings:**

Ethiopia’s transformation was enabled by strong national governance and institutional leadership, the adoption of interoperable standards and a unified health data platform, investments in workforce capacity through mentorship and university partnerships, and the introduction of innovative digital services such as electronic community health information system and electronic logistics systems. These initiatives improved data quality, expanded digital service delivery and supported evidence-based decision-making. However, persistent challenges include resource constraints, limited digital literacy among frontline workers, and heavy reliance on donor financing.

**Conclusions & Significance:**

Ethiopia’s nationally led and capacity-focused approach demonstrates that sustainable digital health transformation in LMICs requires more than technology; it demands governance reform, local ownership and long-term financing strategies. The lessons from Ethiopia provide transferable insights for other countries seeking to build resilient, self-reliant digital health ecosystems.

## INTRODUCTION AND PURPOSE

Across the globe, digital health is gaining momentum as a transformative vehicle in reimagining how health systems deliver equitable, efficient and people-centered care. Particularly in low- and middle-income countries (LMICs), the shift from fragmented, donor-driven interventions to locally led, sustainable digital health ecosystems is becoming a defining trend. Central to this shift is *local capacity strengthening*—the development of in-country expertise, institutions and governance structures that can independently design, implement and sustain digital health innovations.

For over a decade, Ethiopia has been on a determined journey to modernize its healthcare system, strategically leveraging digital technology as a cornerstone of transformation. This national move is a direct response to the fundamental need for accessible, high-quality care and aims to address systemic challenges that have historically constrained health outcomes [1]. The earlier digital health landscape in Ethiopia was characterized by fragmented, donor-driven projects that resulted in data silos, duplication of effort and inefficient use of resources [2].

In response, the Government of Ethiopia shifted its strategy toward localization—a paradigm that empowers national actors, strengthens institutional capacity and ensures alignment with government priorities and community needs [3, 4]. This approach includes embedding technical experts within public health institutions, establishing long-term partnerships with local universities to advance research and workforce development, and creating innovation hubs to design and adapt contextually relevant digital health solutions [3].

By investing in local technical and leadership capacity, Ethiopia has increased national ownership of the digital health agenda, transitioning from externally driven efforts to a sustainable, government-led ecosystem [4, 5]. This case study explores the pre-transformation context, strategic frameworks, governance, core digital systems, achievements, challenges and future directions.

Framed as a descriptive case study, this paper is guided by the following primary research questions:

- What were the primary driving forces and contextual challenges that prompted Ethiopia’s national digital health transformation?
- What have been the key milestones, strategic frameworks and governance structures established to build local digital health capacity?
- What are the principal achievements, persistent challenges and transferable lessons learned from Ethiopia’s experience that can inform similar efforts in other LMICs?

## METHODOLOGY

This paper is structured as a descriptive case study of Ethiopia’s national digital health transformation. The methodology combines a comprehensive review of key documents with the authors’ direct programmatic and policy expertise, which provides a unique validation and contextualization of the findings. This design was adopted, positioned as a policy analysis of Ethiopia’s digital health transformation, rather than hypothesis-driven empirical research.

### Study design

A qualitative descriptive case study approach was used to provide an in-depth, narrative account of the driving forces, processes, achievements and challenges of strengthening digital health local capacity in Ethiopia between 2016 and 2025.

### Data sources

The analysis is based on a wide range of publicly available documents and the authors’ institutional knowledge. Key data sources included:

- **Government strategic documents:** National policies and frameworks such as the Health Sector Transformation Plan (HSTP I, 2016–2020 and HSTP II, 2020 to June 2025), Health Sector Medium Term Development and Investment Plan (HSDIP, 2023–2026), the Information Revolution (IR, 2016–2020 & 2021–2025) Roadmap, the national eHealth Architecture (2018, revised in 2022), the Digital Health Blueprint 2030 (DHBp, 2021–2030), and other strategic and operational protocols.
- **Peer-reviewed literature:** Academic articles and publications focusing on Ethiopia’s health information systems, digital health implementation and health system strengthening.
- **Official reports and publications:** Program evaluations, annual reports and special bulletins from the Ethiopian Ministry of Health, the World Health Organization (WHO), the World Bank, the Global Fund, Gates Foundation and other key development partners.
- **Project and implementation documents:** Publicly accessible reports, policy briefs and case studies from major implementing partners, including John Snow Inc. (JSI) and the United Nations Development Program (UNDP).
- **Experiential data:** As active participants in Ethiopia’s digital health ecosystem, the authors from both the Ministry of Health and JSI drew upon their direct operational, M&E and policy experience to validate the findings from the document review, particularly concerning the achievements, innovations and persistent challenges described.

### Data collection and selection

Documents were systematically selected based on their relevance to Ethiopia’s national digital health strategy and local capacity-building efforts. The inclusion criteria were: (1) documents published between 2015 and 2025; (2) focus on national-level digital health strategy, governance, systems and outcomes in Ethiopia and (3) originating from credible sources such as government bodies, academic journals, UN agencies or primary implementing partners. Documents focusing on isolated, non-scalable pilot projects without national strategic linkage were excluded.

### Data analysis

A thematic content analysis approach was used to synthesize the collected data. Information from the documents was extracted and organized according to pre-defined and emergent themes aligned with the paper’s core objectives: the pre-transformation context, strategic frameworks, governance, core digital systems, achievements, challenges and future trajectory. A timeline analysis was employed to structure the narrative chronologically, illustrating the evolution of the digital health ecosystem. The synthesis of these findings was collaboratively reviewed and validated by all authors to ensure accuracy and provide a comprehensive, grounded analysis of Ethiopia’s journey.

## GLOBAL CONTEXT OF DIGITAL HEALTH TRANSFORMATION

Ethiopia’s journey is reflective of a broader global movement among LMICs to harness digital technology for health system strengthening [6]. Globally, there is a noticeable shift from fragmented, donor-driven pilot projects toward nationally-owned, scalable digital health ecosystems [7]. This trend is championed by international bodies like the World Health Organization (WHO), which advocates for integrated national eHealth strategies as essential components for achieving Universal Health Coverage (UHC) [8].

Experiences from other countries offer valuable parallels. For instance, Indonesia’s success has been attributed to strong government leadership and policy integration [9], while countries like Tanzania have made strides in interoperability through shared health information exchanges [10]. Similarly, large-scale initiatives in Asia, such as India’s national digital health mission, have demonstrated the potential of digital identity and mobile platforms to expand service delivery [11]. However, common challenges persist across many LMICs, including inadequate infrastructure, shortages of a digitally skilled workforce, and the critical question of long-term financial sustainability [12]. Within this global landscape, the emphasis on strengthening local capacity and governance has emerged as a critical determinant of success for sustainable digital health implementation [13].

## CONTEXT: THE PRE-TRANSFORMATION HEALTHCARE LANDSCAPE

Before 2017, Ethiopia’s healthcare system was burdened by significant structural and operational challenges that impeded the delivery of high-quality health services. The digital health landscape was particularly fragmented, marked by a proliferation of uncoordinated initiatives, many of which were driven by external donors and development partners [2]. This lack of coordination often resulted in duplicative efforts and the deployment of incompatible systems. A striking example was the parallel use of multiple versions of the electronic Health Management Information System (eHMIS), which rendered national-level data aggregation and analysis nearly impossible [14].

The dominance of paper-based systems further compounded problems, as they were frequently characterized by incomplete, inconsistent and untimely data [15, 16]. Compounding this was the inadequacy of digital infrastructure, with frequent power outages and a lack of functioning hardware hampering the use of digital tools [15]. Ethiopia also faced a severe shortage of health professionals with digital skills, with a health workforce-to-population ratio among the lowest globally [1]. Further, a high degree of donor dependency undermined national ownership; in 2016/17, for instance, more than half of Ethiopia’s primary healthcare funding came from external sources [2]. These systemic weaknesses compromised clinical decision-making and limited equitable access to essential services [4, 17].

## FINDINGS: THEMATIC ANALYSIS OF ETHIOPIA’S TRANSFORMATION

Our analysis of Ethiopia’s digital health journey is structured around five predefined themes: (1) governance and institutional leadership; (2) interoperability and standards; (3) workforce capacity development; (4) innovation and service delivery impact; and (5) financing and sustainability.

### Governance and institutional leadership

Strong national leadership from the Ministry of Health has been the central pillar of Ethiopia’s transformation. This was institutionalized through the Health Sector Transformation Plan (HSTP) [18] and its cornerstone, the Information Revolution (IR) [19], which provided the strategic vision for a data-driven health system. To execute this vision, the Ministry established dedicated executive offices and coordinating bodies to oversee digital health initiatives, ensuring alignment with national policies and effective multi-stakeholder coordination [16, 20]. This centralized governance was instrumental in shifting from fragmented, donor-driven projects to a coherent national strategy [2]. A key governance tool introduced was the Digital Health Project Inventory System (DHPIS), a national registry and certification platform functioning as a clearinghouse to regulate digital tools and prevent duplication [20].

### Interoperability and standards

From the outset, Ethiopia prioritized interoperability. The country’s first national eHealth Architecture (eHA) (2018) served as the technical blueprint for system integration, standardization and secure data exchange [21]. This effort was guided by the Digital Health Blueprint 2021–2030 [22] and the broader national Enterprise Architecture (EA) framework [23]. To operationalize this, the Ministry of Health has embarked on developing its own sector-specific Health EA, a process now significantly advanced. Foundational elements were established to support this ecosystem, including the Master Facility Registry (MFR), the National Health Data Dictionary (NHDD) for data consistency, and a Product Catalog to enable supply chain interoperability [24]. Ethiopia also adopted HL7 FHIR for data exchange and began transitioning to ICD-11, reflecting a commitment to global standards [1, 25]. A landmark achievement in standardization was the migration to a single, unified DHIS2 platform. Its integration with the Master Facility Registry (MFR) served as the nation’s first successful proof of concept for large-scale digital health interoperability, enabling a seamless rollout to ˃30 000 facilities and achieving reporting completeness rates exceeding 90% [14, 26]. Demonstrating a commitment to broader national digitization, the health sector is also strengthening inter-agency coordination to leverage Digital Public Infrastructure (DPI), such as digital payment systems and the national ID initiative [39]. Most recently, the Ministry of Health and its partners have been ideating the concept of an ‘Interoperability Highway’ and have begun the journey to materialize it. The initiative is designed to enhance data exchange across systems by replacing the current point-to-point integrations, which are not as scalable or efficient as required.

### Workforce capacity development

Recognizing that investing in people is as important as investing in technology, Ethiopia made sustained efforts to build a digitally literate health workforce. A flagship initiative, the Capacity Building Mentorship Program (CBMP), run in partnership with universities, enhanced data quality and analytical skills among health professionals [16]. The Health Information Technology (HIT) Internship Program further created a pipeline of professionals capable of managing and scaling systems [27]. Despite progress, challenges remain: frontline workers still report concerns about workload, limited digital confidence and resistance to new technologies [36]. Addressing these human factors through continuous training, mentorship and user-centered design remains a critical priority. Looking ahead, the very recent ratification of Ethiopia’s Start-up Act (July 2025) offers a timely opportunity to boost vibrant start-ups as gap-fillers in the health sector, complementing workforce capacity by fostering innovative, agile solutions where institutional mechanisms may fall short.

### Innovation and service delivery impact

Ethiopia’s digital transformation translated into innovative services and measurable healthcare impacts. The electronic Community Health Information System (eCHIS) digitized services for over 25 000 Health Extension Workers (HEWs), improving maternal and child health services for an estimated 22 million people [28]. Digitally enabled performance incentives through eCHIS increased HEW performance scores from 66 to 82 [29]. At higher levels, Electronic Medical Records (EMRs) were deployed in over 70 high caseload facilities [30], while the electronic Logistics Management Information System (eLMIS) enhanced supply chain visibility [31]. The country has also piloted telemedicine to connect rural facilities with specialists [32] and explored AI-powered decision support tools for HEWs [33]. Collectively, these efforts contributed to improved health outcomes, including reductions in maternal and child mortality during the Health Sector Transformation periods [34]. While these examples showcase significant progress, they represent only a fraction of the innovation underway. To sustain and accelerate this impact, it is essential for the government to foster a collaborative ecosystem, actively embracing novel solutions from the private sector, academia and global partners in the years to come.

### Financing and sustainability

Long-term sustainability remains a major challenge. Ethiopia’s health system has historically relied heavily on donor funding, which accounted for more than half of primary healthcare financing in 2016/17 [2]. Current domestic spending on digital health covers ˂4% of needs, highlighting a major funding gap [35]. To address this, Ethiopia’s strategy emphasizes innovative domestic financing models and greater private-sector participation [37]. Initiatives such as inclusive digital health payment solutions aim to mobilize domestic resources and reduce dependence on external funding, thereby ensuring resilience and sustainability [38].

## DISCUSSION

Ethiopia’s digital health transformation provides a compelling case study for LMICs. The journey has yielded valuable insights while also underscoring persistent challenges. One of the most significant lessons is the critical importance of national leadership in overcoming fragmentation [2]. Another key takeaway is the need to prioritize interoperability from the outset (‘Interoperability by Design’) through a national architecture and adoption of global standards [21, 25]. Moreover, the country’s experience highlights that investing in people through programs like the CBMP is as important as investing in technology [16]. Despite advances, challenges remain, including significant resource constraints, with public sector spending covering ˂4% of the estimated funding need [35]. User adoption is another barrier, as frontline workers express concerns about increased workload and low digital confidence [36]. Finally, ensuring long-term sustainability by developing viable domestic financing mechanisms to reduce donor reliance is essential for sustaining gains [2, 37].

## STUDY LIMITATIONS

This study has several limitations. First, the analysis relies on secondary data, which may not capture the nuanced perspectives of frontline workers. Second, there is a potential for bias in source materials, as government and partner-led reports may inherently emphasize successes. The authors’ insider knowledge helps validate findings but also carries a risk of bias. Finally, as a case study of Ethiopia, the findings have limited generalizability, as the country’s unique context means that specific strategies may not be directly transferable without significant local adaptation. Future research could triangulate these findings with primary data from frontline workers and comparative studies across countries to strengthen external validity.

## CONCLUSION

Ethiopia’s digital health transformation, anchored in the strategic vision of its Information Revolution, offers a compelling model for other LMICs. Through deliberate efforts in governance reform, systems development and local capacity strengthening, the country has made measurable progress in reshaping its health information landscape. Notable achievements include the implementation of interoperable national data systems, the institutionalization of data-driven decision-making, and the expansion of citizen-centered digital health services.

Nonetheless, significant challenges remain, including resource constraints and questions around long-term financial sustainability. By addressing these challenges head-on, Ethiopia is not only advancing toward its national health goals but also emerging as a leader in the digital health space, offering actionable lessons for others seeking to build sustainable, government-led digital health ecosystems.

## Data Availability

The data that support the findings of this study are available from the corresponding author upon reasonable request.
